# Supplementary material for: Mapping and rewiring the MYBPC3 promoter for rescue of haploinsufficiency driven hypertrophic cardiomyopathy
Source: bioRxiv. 2026 Sep 22:2026.09.20.752987. Preprint. [Version 1] doi: 10.64898/2026.09.20.752987 (PMC13622176; doi:10.64898/2026.09.20.752987)
Supplement: Supplementary file 1 [file NIHPP2026.09.20.752987v1-supplement-3.pdf]

bioRxiv preprint doi: https://doi.org/10.64898/2026.09.20.752987; this version posted September 22, 2026. The copyright holder for this preprint (which was not certified by peer review) is the author/funder, who has granted bioRxiv a license to display the preprint in perpetuity. It is made available under a CC-BY 4.0 International license.

Supplemental Figures

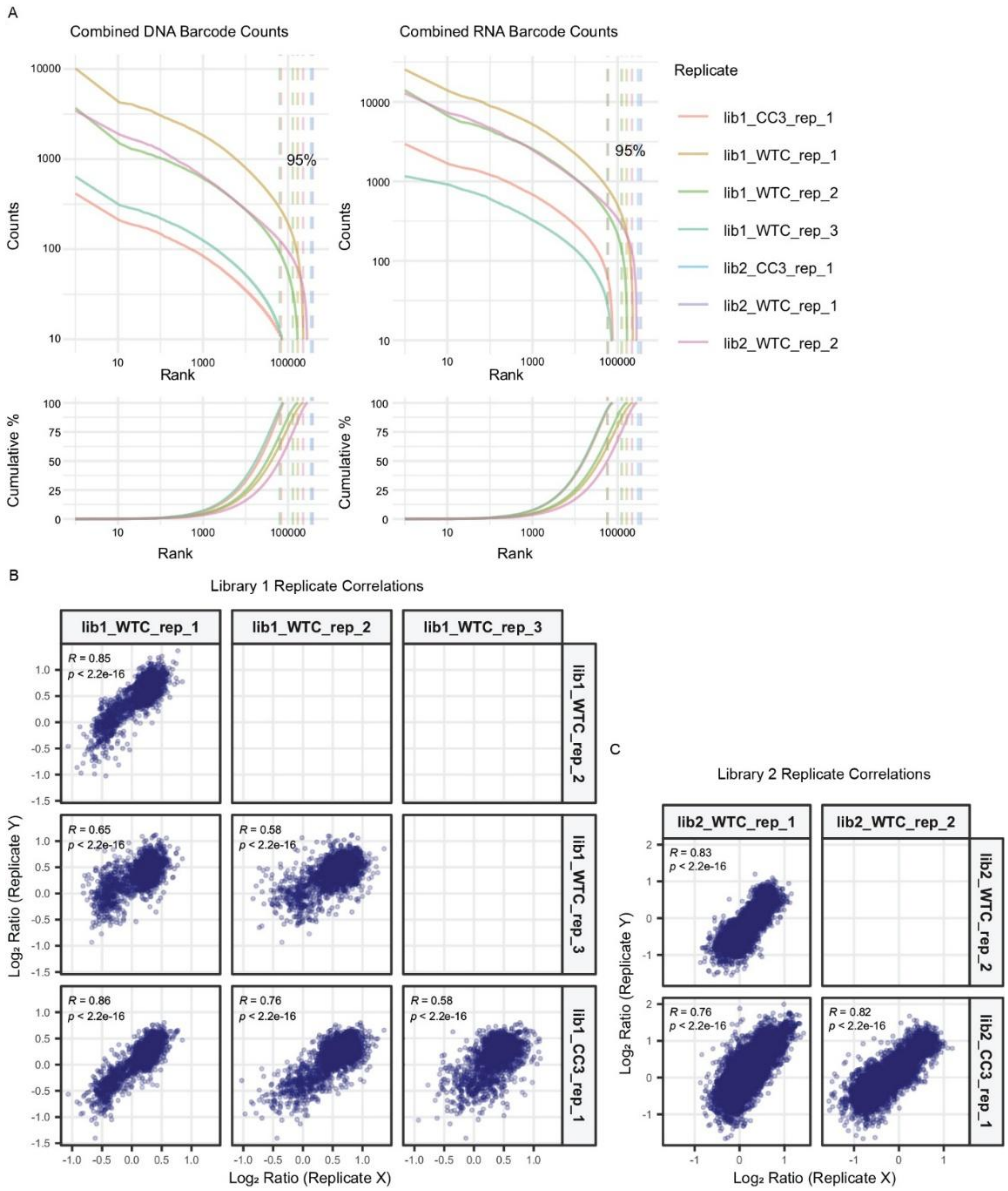

Figure S1: Waterfall plots and replicate correlations for MPRA. The MPRA library 1 consisted of SNVs for *MYBPC3* promoter positions 211-456, as well as rolling triplet variants and *TNNT2-MYBPC3* hybrid promoters. Library 2 consisted of SNVs for *MYBPC3* promoter positions 1-210, combinations of SNVs in positions 211-456, as well as *MYBPC3* hybrid promoters and synthetic enhancers. **(A)** 95% of barcodes had high DNA and RNA counts. Barcodes with fewer than 10 counts in a given replicate were excluded from analysis. Library 1 **(B)** and library 2 **(C)** Pearson correlations demonstrate high concordance of variant mean DNA/RNA ratio data across biological replicates.

bioRxiv preprint doi: https://doi.org/10.64898/2026.09.20.752987; this version posted September 22, 2026. The copyright holder for this preprint (which was not certified by peer review) is the author/funder, who has granted bioRxiv a license to display the preprint in perpetuity. It is made available under a CC-BY 4.0 International license.

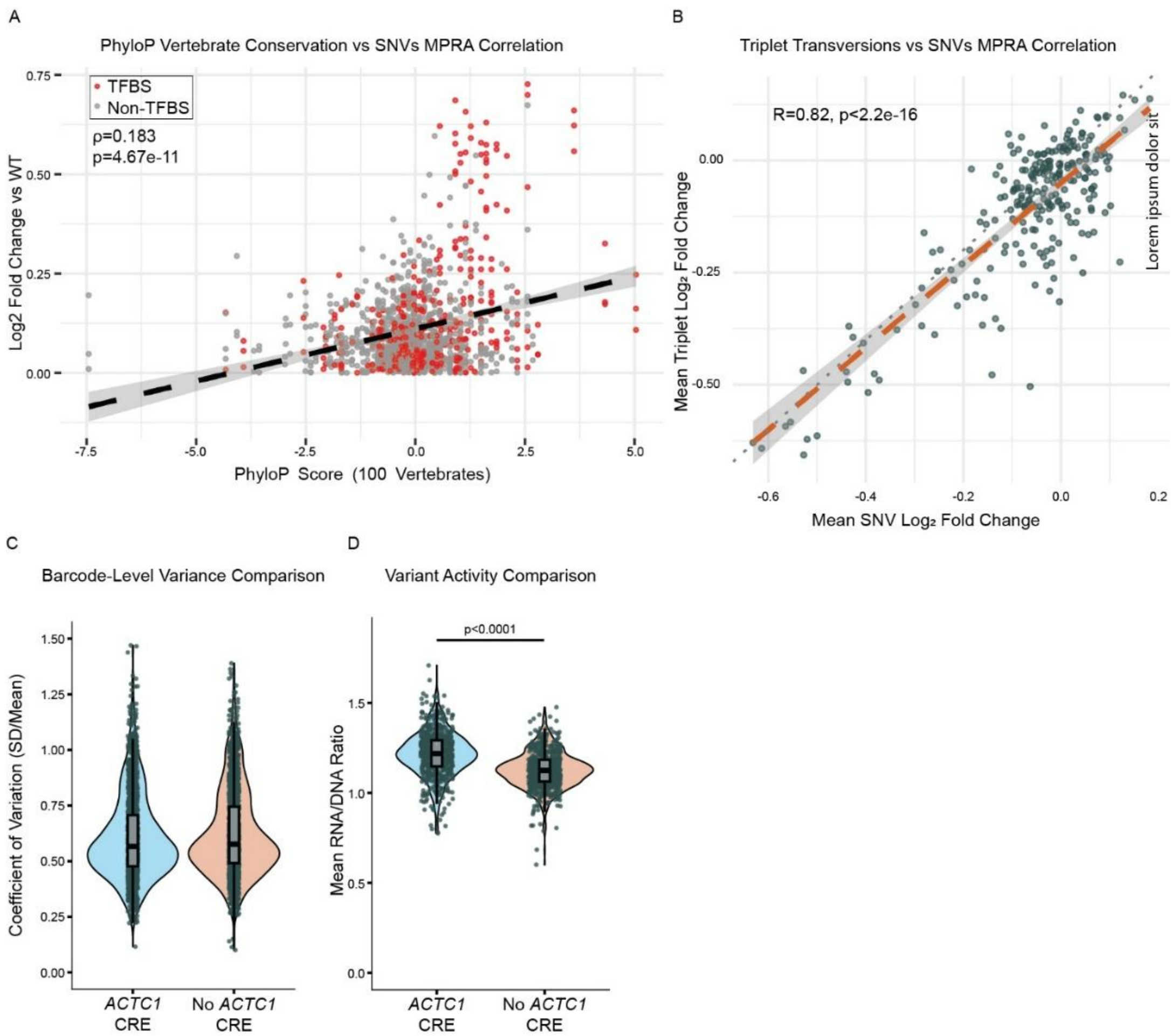

Figure S2: Analysis of alternative approaches to MPRA library construction. (A) Vertebrate conservation is only very weakly correlated with SNV MPRA effect size (Spearman correlation). **(B)** Rolling triplet transversions represent an alternative to saturation mutagenesis for TFBS mapping through MPRA, trading mapping resolution for the ability to cover the same genomic territory with decreased library complexity, thus requiring fewer cells for each experimental replicate. Impact on activity for each position was highly correlated between the triple variants and SNVs. (Pearson correlation) **(C-D)** A portion of the library (SNVs for positions -428 to -219) was designed with paired duplicates lacking the upstream ACTC1 enhancer to test if that enhancer improved signal:noise in the MPRA. While versions with the enhancer increased mean barcode activity (mean RNA/DNA ratio = 1.22 with enhancer vs 1.13 without, $p<0.0001$ Wilcoxon test), it did not improve the coefficient of variation in a biologically meaningful way (CV=0.577 with enhancer vs 0.566 without, $p<0.01$ Wilcoxon test).

bioRxiv preprint doi: https://doi.org/10.64898/2026.09.20.752987; this version posted September 22, 2026. The copyright holder for this preprint (which was not certified by peer review) is the author/funder, who has granted bioRxiv a license to display the preprint in perpetuity. It is made available under a CC-BY 4.0 International license.

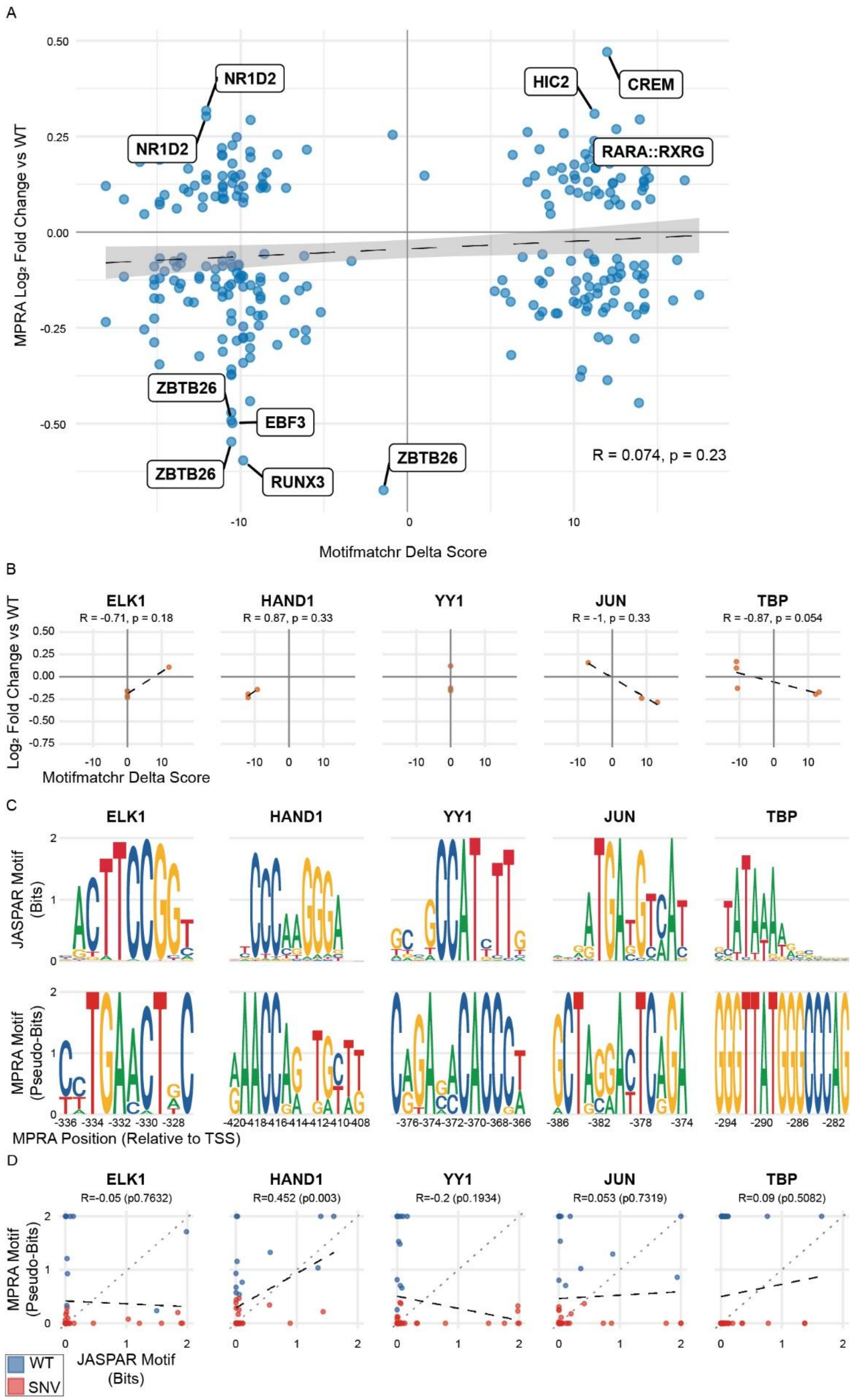

bioRxiv preprint doi: https://doi.org/10.64898/2026.09.20.752987; this version posted September 22, 2026. The copyright holder for this preprint (which was not certified by peer review) is the author/funder, who has granted bioRxiv a license to display the preprint in perpetuity. It is made available under a CC-BY 4.0 International license.

Figure S3: Motifmatchr analysis of non-annotated SNVs and additional annotated TFBSs. (A) Predicted SNV impact on TFBS binding affinity (Delta Score) was calculated using motifmatchr[16] (see Methods) and compared to impact on transcription in the MPRA for SNVs outside of TFBS annotations (Pearson correlation). **(B)** Correlation of predicted SNV impact on binding efficiency compared to impact on MPRA transcription (Pearson correlation). Additional annotated TFBSs not shown in **Figure 2C**. Positive correlation indicates that TF is acting as an activator while negative correlation suggests it is acting as a repressor. **(C)** Logo-style motifs for TFBSs were generated empirically from MPRA SNV data by calculating a contribution score for each base at each position (see Methods) and are shown beneath their canonical motifs from JASPAR[17]. Additional TFBSs not shown in **Figure 2D**. **(D)** The contribution score for each base calculated in generating the logo-style motifs was compared to the contribution scores from the canonical JASPAR motif (Pearson correlation). Additional TFBSs not shown in **Figure 2E**.

bioRxiv preprint doi: https://doi.org/10.64898/2026.09.20.752987; this version posted September 22, 2026. The copyright holder for this preprint (which was not certified by peer review) is the author/funder, who has granted bioRxiv a license to display the preprint in perpetuity. It is made available under a CC-BY 4.0 International license.

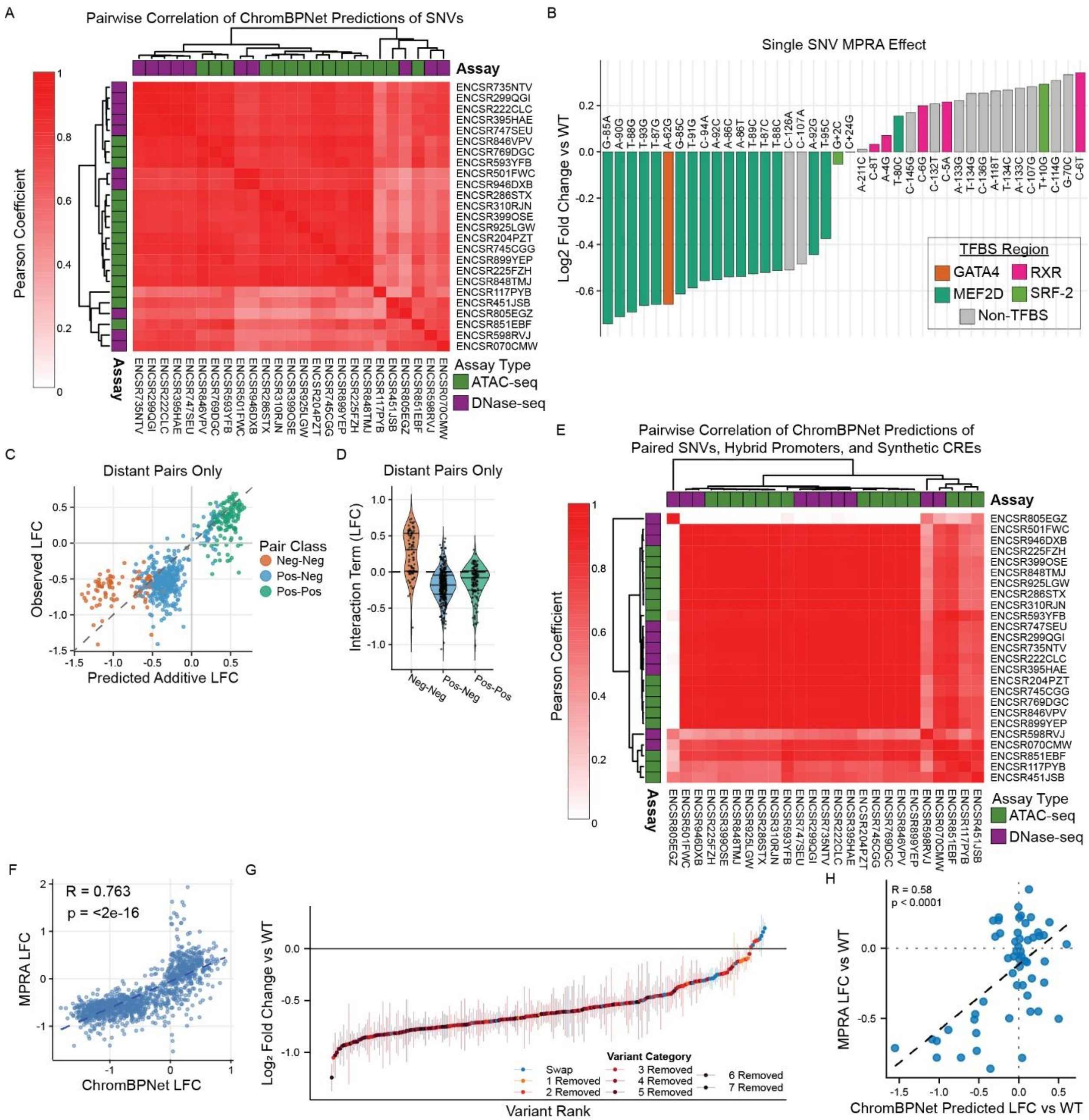

Supplemental Figure S4: ChromBPNet model pairwise correlation, paired SNVs, and additional tested hybrid promoters. (A) 25 ChromBPNet models were trained on different DNase and ATAC-seq datasets from human left ventricle samples. Pairwise analysis of these models shows high correlation on the simulated ATAC-seq read counts predicted for each *MYBPC3* promoter SNV variant sequence in the MPRA libraries (mean Fisher z-transformed r = 0.76, 95%CI = [0.76-0.77], mean p value < 0.00001). **(B)** LFC_{WT} of the SNVs with the 20 largest positive and 20 largest negative MPRA effects that were selected for the paired SNV analysis.

bioRxiv preprint doi: https://doi.org/10.64898/2026.09.20.752987; this version posted September 22, 2026. The copyright holder for this preprint (which was not certified by peer review) is the author/funder, who has granted bioRxiv a license to display the preprint in perpetuity. It is made available under a CC-BY 4.0 International license.

(C) A weighted linear model was created for each possible pair among these 40 variants using the single SNV LFC_{WT} to predict the paired LFC_{WT} assuming additive effects (**Figure 3C**). In an alternate model, excluding pairs of SNVs that are within 10bp of each other and presumably affect the same TFBS did not meaningfully change the correlation of LFC_{WT} predicted by the weighted linear model compared to observed MPRA LFC_{WT} ($R = 0.80$, $p < 0.0001$, Pearson correlation). **(D)** This alternate model has the same pattern of sub-additivity in each category seen in the fully inclusive model. **(E)** Pairwise analysis of the ChromBPNet predictions for paired SNVs (**Figure 3C**), hybrid *MYBPC3* promoter elements (**Figure 3H**), and synthetic CREs (**Figure 4E**). The 25 models trained were highly concordant (mean pairwise Fisher z-transformed r = 0.88, 95%CI = [0.87,0.89], mean p-value = 0.0098). **(F)** The ChromBPNet models were highly correlated with the MPRA data. ($r = 0.763$, $p < 0.0001$) **(G)** The *MYBPC3* promoter was divided into 8 elements (**Figure 3H**). Each combination of 1, 2, 3, 4, 5, 6, and 7 elements were removed and replaced with randomized sequence. Each element was also swapped for each other element. These variants were compared in an MPRA to the WT promoter. **(H)** ChromBPNet was moderately correlated with MPRA data for the hybrid promoters but tended to underpredict promoters that increased expression in the MPRA ($r = 0.58$, $p<0.0001$).

Significance: *FDR<0.05, **FDR<0.01, ***FDR<0.001, Wilcoxon rank sum test with Benjamini-Hochberg correction and 95%CI unless otherwise noted.

bioRxiv preprint doi: https://doi.org/10.64898/2026.09.20.752987; this version posted September 22, 2026. The copyright holder for this preprint (which was not certified by peer review) is the author/funder, who has granted bioRxiv a license to display the preprint in perpetuity. It is made available under a CC-BY 4.0 International license.

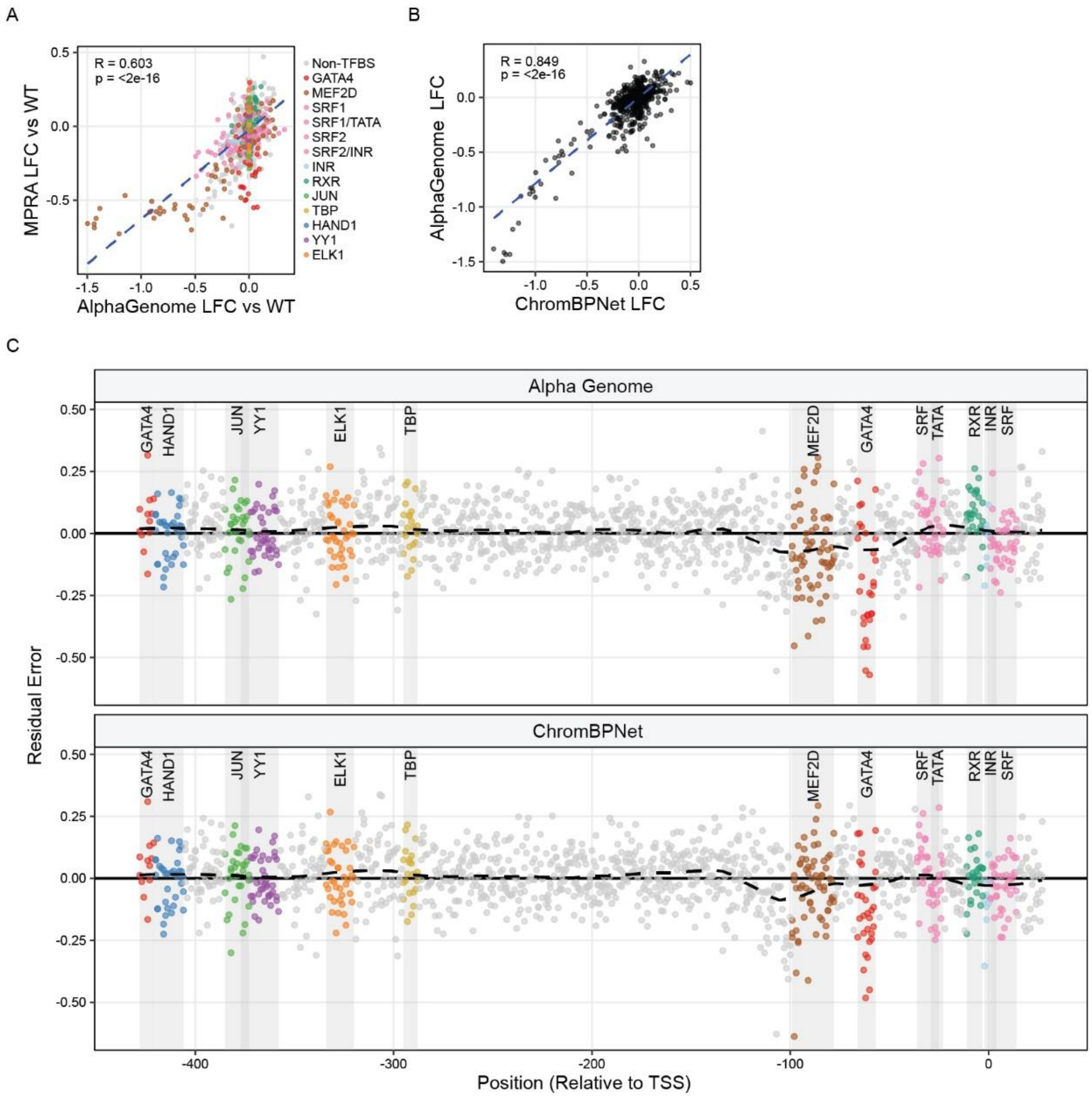

Supplemental Figure S5: AlphaGenome predicted SNV effect on expression similarly to ChromBPNet. (A) AlphaGenome was used to perform in silico saturation mutagenesis of the *MYBPC3* promoter with 150kb of context. The predicted effects of SNVs on expression were moderately but significantly correlated with the MPRA experiment data (r = 0.60, p<0.0001). **(B)** AlphaGenome predictions were highly correlated with the ChromBPNet predictions for corresponding variants (r = 0.85, p<0.0001). **(C)** Analysis of the scale-adjusted residual error from the correlation of the two models with the MPRA data shows similar patterns of over and under predictions throughout the promoter sequence.

bioRxiv preprint doi: https://doi.org/10.64898/2026.09.20.752987; this version posted September 22, 2026. The copyright holder for this preprint (which was not certified by peer review) is the author/funder, who has granted bioRxiv a license to display the preprint in perpetuity. It is made available under a CC-BY 4.0 International license.

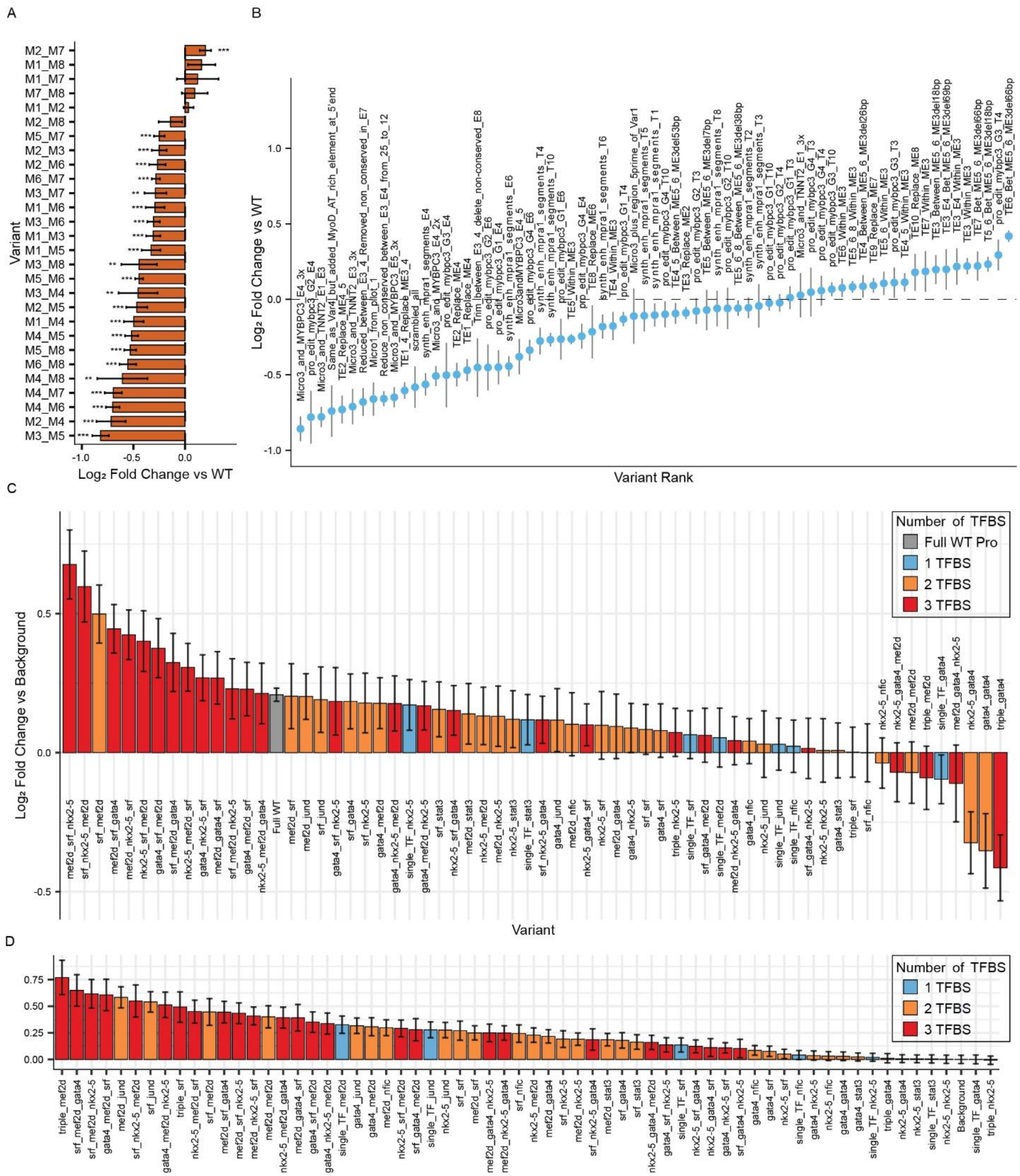

Supplemental Figure S6: Hybrid promoters and synthetic CREs can drive transcription.

(A) Effects of each element swap variant on transcription. **(B)** 68 *TNNT2* and *MYBPC3* hybrid promoters were tested in an MPRA and compared to the full WT *MYBPC3* promoter. **(C)** All

bioRxiv preprint doi: https://doi.org/10.64898/2026.09.20.752987; this version posted September 22, 2026. The copyright holder for this preprint (which was not certified by peer review) is the author/funder, who has granted bioRxiv a license to display the preprint in perpetuity. It is made available under a CC-BY 4.0 International license.

synthetic CREs tested in the MPRA compared to the null background sequence. **(D)** ChromBPNet models predicted the $LFC_{Background}$ for the same homo- and heterotypic TFBS clusters.

Significance: *FDR<0.05, **FDR<0.01, ***FDR<0.001, Wilcoxon rank sum test with Benjamini-Hochberg correction and 95%CI unless otherwise noted. Mean $LFC_{Background}$ and 95%CI calculated across all replicates and barcodes for each variant for MPRA data, and calculated across the 25 models for ChromBPNet.

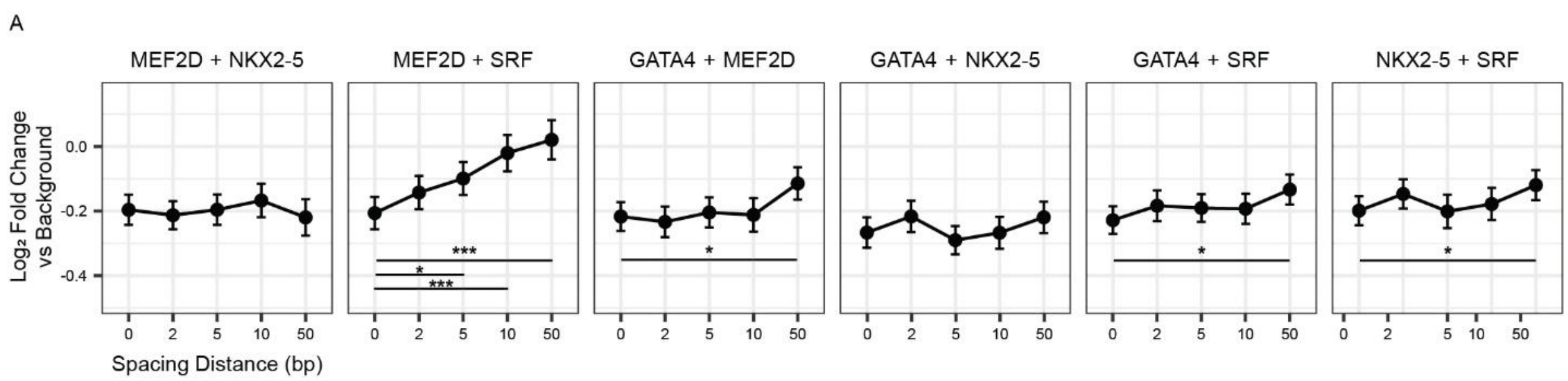

Supplemental Figure S7: Impact of TFBS spacing on transcriptional strength.

(A) Effect of spacing between two TFBSs within the null sequence for homotypic and heterotypic pairs was assessed in the context of the minimal *MYBPC3* promoter. Spacing intervals of 0, 2, 10, and 50 bp were measured, heterotypic pairs shown here (homotypic pairs are shown in **Figure 4H**).
